# Deletion of fbiC in Streptomyces venezuelae removes autofluorescence in the excitation-emission range of cyan fluorescent protein

**DOI:** 10.1099/mic.0.001552

**Published:** 2025-04-15

**Authors:** Parminder Singh Mavi, Klas Flärdh

**Affiliations:** 1 Department of Biology, Lund University, Kontaktvägen 13, 223 62 Lund, Sweden

**Keywords:** *Actinomycetota*, autofluorescence, F420, *Streptomyces*

## Abstract

Autofluorescence poses an impediment to fluorescence microscopy of biological samples. In the Gram-positive, soil-dwelling bacteria of the genus *Streptomyces*, sources of autofluorescence have not been examined systematically to date. Here, we show that the model organism for the genus, *Streptomyces venezuelae*, shows autofluorescence in two of the commonly used fluorescence channels for visualizing cyan and green/yellow fluorescent proteins. We identify the source of autofluorescence in the cyan fluorescence channel as redox cofactor factor 420 (F_420_) and target its synthesis to remove it. By deleting the *vnz15170* (*fbiC*) gene, which is a key biosynthetic gene for the production of F_420_, we were able to create an autofluorescence-free strain in the cyan range of fluorescence excitation-emission. We demonstrate the usefulness of this strain by imaging the mTurquoise-tagged polar growth-related protein DivIVA and the cell division-related protein FtsZ in the *fbiC* deletion background. Using live-cell imaging to follow the dynamics of DivIVA and FtsZ, we demonstrate an improved signal-to-noise ratio in the mutant strain. We show that this strain can be a suitable tool for visualizing the localization of proteins in *Streptomyces* spp. and can facilitate the utilization of multi-colour imaging and fluorescence resonance energy transfer-based imaging.

## Data Summary

Supplementary material is available with the online version of this article, available through Figshare at 10.6084/m9.figshare.28430591 [1]. The datasets used and/or analysed during the current study are available from the authors upon request.

## Introduction

Autofluorescence is defined as the intrinsic fluorescence of cells and tissues. Biological samples autofluoresce under certain wavelengths of electromagnetic radiation due to endogenous fluorescent properties [2,4]. This can arise from fluorescent molecules like flavins [5], NAD(P) [6], aromatic amino acids [7] or structural proteins, such as collagen [8]. Bacteria can exhibit autofluorescence arising from several of these sources. For example, *Rhodospirillum rubrum* exhibits autofluorescence in the near-infrared region from the presence of bacteriochlorophylls [9]. Spores of *Bacillus subtilis* exhibit autofluorescence characteristic of flavin, which has been exploited for fluorescence-based detection of spores in aerosols [10,11]. Bacteria forming dental plaques exhibit strong autofluorescence, hypothesized to originate from protoporphyrin IX [12]. Autofluorescence in mycobacteria arising from factor 420 (F_420_) has been noted [13,15] and utilized as a diagnostic tool for detecting and studying these bacteria in environmental samples and clinical specimens. While bacterial autofluorescence has been exploited to study cellular structures, metabolic activities and disease processes without the requirement for external fluorophores [13,16,18], it poses impedance to advanced fluorescence microscopy when fluorescent tags are used for the study of localization and dynamics of subcellular components [19].

Gram-positive bacteria of the group streptomycetes inhabit soil environments and undergo a complex developmental cycle marked by morphological transformations from a multi-genomic, nutrient-scavenging mycelium to dormant, unigenomic spores [20,21]. The genomes of these bacteria are relatively large and contain diverse biosynthetic gene clusters for the synthesis of a plethora of secondary metabolites, which sometimes result in autofluorescence in the bacteria. For example, red autofluorescence in *Streptomyces coelicolor* has been reported earlier and has been attributed to prodiginine pigment production [16]. Additionally, certain antibiotics produced by various *Streptomyces* spp. show strong fluorescence. Tetracyclines, for instance, are intrinsically fluorescent, and this has been exploited to assay antibiotic production in fermentation. Other noteworthy examples of fluorescent antibiotics are filipin [22] and plicamycin [23] isolated from *Streptomyces filipinensis* and *Streptomyces plicatus*, respectively. Moreover, apart from secondary metabolite-sourced autofluorescence, model organisms from this genus exhibit characteristic punctate autofluorescence within the green/yellow fluorescence range [24,25].

The presence of autofluorescence in *Streptomyces* spp. is problematic when applying advanced fluorescence imaging to the bacteria. *Streptomyces venezuelae* is one of the main model organisms for studying the cell and developmental biology of this metabolically diverse genus. The bacterium exhibits many advantageous characteristics, such as fast growth and the ability to complete its life cycle in a liquid medium [26,27]. This is particularly advantageous for live cell imaging, where both vegetative and sporulating stages can be examined [26]. With its increasing application to cell biological studies and the ever-expanding toolbox of fluorescent labels, the issues of autofluorescence in the strain need to be addressed to allow better imaging possibilities. Here, we examine the autofluorescence in *S. venezuelae* with the three commonly utilized fluorescence filter combinations for the visualization of cyan, yellow and red fluorophores for fluorescence imaging. We identify and dissect the cause of autofluorescence in the cyan region of the spectrum. Through rational genetic manipulation, we successfully engineered a strain of *S. venezuelae* with significantly reduced autofluorescence, specifically in the cyan fluorescence range. This strain widens the toolbox of fluorescent tags available for imaging in *Streptomyces* by accommodating multi-colour imaging.

## Methods

### Bacterial strains and growth conditions

The bacterial strains used in this study are listed in Table S1 (available in the online Supplementary Material). *S. venezuelae* strain NRRL B-65442 was used for all deletions and genetic modifications. Cultivation of *S. venezuelae* was carried out at 30 °C and in maltose yeast extract medium (MYM) supplemented with trace elements [28]. For interspecies conjugation, soy flour mannitol medium was used, as described previously [29]. The antibiotics apramycin (50 µg ml^−1^), nalidixic acid (20 µg ml^−1^) and hygromycin (50 µg ml^−1^) were used where required, as described [29]. *Escherichia coli* strain ET12567/pUZ8002 was used as the donor strain for conjugation to *S. venezuelae. E. coli* strain DH5α was used for cloning, modification and maintenance of plasmids.

### Bioinformatic analyses

Bioinformatic prediction of the domains of FbiC was done using InterproScan (Expasy) [30]. Kyoto Encyclopedia of Genes and Genomes (KEGG) pathways were used for finding genes related to the production of coenzyme F_420_ [31,33] in *S. venezuelae*. The Integrative Genomics Viewer was used to visualize the transcription start site data of *S. venezuelae* [34].

### Gene modification, cloning and plasmids

Q5 or Taq DNA polymerase (NEB) was used for all PCR amplification reactions. Oligonucleotides used for PCR and cloning are listed in Table S2 and the plasmids in Table S3. Lambda Red-mediated recombineering was used for the deletion of *fbiC* and replacement with an apramycin resistance marker (*apra*), using *E. coli* strain DY380 for modification of cosmids for gene deletion [35]. Primers KF1764 and KF1765 were used for the amplification of the *apra* cassette from the plasmid pIJ773 and recombineering of cosmid pKF763 for creating pKF824. *E. coli* strain ET12567/pUZ8002 was used for the mobilization of plasmids and cosmids containing *oriT* into *S. venezuelae*, as described previously [36]. *E. coli* strain BT340 expressing yeast Flp recombinase [37] was used for excision of *FRT*-flanked deletion cassette from pKF824 and modified with the addition of hygromycin resistance cassette (*hyg*) to create pKF962. For verification of the gene deletions, PCR was performed with the diagnostic primers KF2059/KF2060 or KF2059/KF2061. pKF717 was created by ligating *mTq2* (encoding mTurquoise2) amplified from codon-optimized sequence in pmTurquoise2 for expression in *S. venezuelae* using primers KF1717 and KF1718 in pSS204 (digested with KpnI and XhoI).

### Fluorescence microscopy and live imaging

Fluorescence imaging was performed on either Zeiss AxioObserver.Z1 microscope with Illuminator HXP 120 V lamp (Zeiss) or on Nikon Ti microscope (Nikon) using CellASIC ONIX2 microfluidics system (Merck), as described earlier [38]. For imaging the localization of mTq2-tagged DivIVA and examining autofluorescence in the WT and the mutant strains, a Zeiss Axio Observer.Z1 with a temperature-controlled cage incubator was used with phase contrast imaging and widefield fluorescence microscopy. The objective used was Plan-Apochromat 100 ×/1.4 Oil Ph3 objective and phase contrast setting. Fluorescence imaging was done with an HXP 120 V light source and excitation filter range of 424–448 nm and an emission filter set for 460–500 nm. Spores were seeded and maintained at 30 °C using the stage cage incubator. For vegetative growth only, the MYM medium was used, while for sporulation, after 6 h of growth with the MYM medium, the inflow was switched to the spent MYM medium, as described previously [26]. Imaging was done at intervals of 15 min. Images were captured using the ORCA Flash 4.0 LT camera (Hamamatsu). For examination of the localization of FtsZ-mTq2, the Nikon Ti Microscope was used with differential interference contrast (DIC) bright field imaging. The objective lens used was Apo TIRF 100× DIC oil. DIC imaging was performed with an LED DIA-Illuminator (3.0 V). The light source used was Nikon MultiLaser (LU4A) 405 nm (power 9). For sporulation, the protocol was used as described by Schlimpert *et al*. [26], with spent MYM used from the *fbiC* mutant strain for imaging of the *fbiC* mutant strain and WT in the case of the WT derivative strain. Images were captured with the Prime 95B CMOS camera. FM4-64 staining of the cell membrane was performed in homemade growth chambers [39] by adding the dye to the concentration of 0.5 µg ml^−1^ in a MOPS minimal medium with glucose as the carbon source. Cells were incubated at 30 °C for 8 h and then imaged using the Nikon Ti Eclipse microscope with the same settings as for DivIVA-mTq2 and using the FM4-64 filter for the cell membrane staining.

### Spectrofluorometry

Crude cell lysates of WT and LUV320 strains were prepared by mechanical lysis using a FastPrep-24 bead beater (MP Biomedicals). A 10 ml culture of *S. venezuelae* was grown to an OD_600_ of 1.0 in MYM medium, harvested by centrifugation and resuspended in 1 ml PBS containing protease inhibitor cocktail and mixed with 500 µl of 0.1 mm zirconia beads placed in a 2 ml cryovial. Bead beating was done at 6 m s^−1^ for 30 s four times with 5 min rest on ice in between. Tubes were then spun at 4 °C and 20,000 ***g*** for 10 min to remove the cell debris. The supernatant was collected, and protein concentration was measured using Bio-Rad DC protein assay [40]. Lysate was normalized for comparison to a total protein concentration of 1 mg ml^−1^ and fluorescence was measured using Cary Eclipse (Agilent) spectrofluorometer with excitation and emission slit width of 5 nm. The emission spectrum was recorded from 430 to 600 nm with an excitation wavelength of 420 nm. The excitation spectrum was measured with emission at 475 nm and excitation range from 380 to 450 nm. For measuring fluorescence from the medium supernatant, MYM medium from cells cultured for the indicated time was separated using centrifugation and filtration. Fluorescence was measured as described above directly on the medium supernatant.

### Disc diffusion sensitivity assay

For the disc diffusion assay to test the redox sensitivity of the *fbiC* strain, MYM agar plates were spread with spores to yield confluent growth (around 100 µl of 10^7^ spores per millilitre), and sterilized 6 mm paper discs soaked either with 5 µl aqueous solution of 1 M diamide or 1 M dithiothreitol (DTT) were placed on the plate. As a control, 5 µl of water was loaded on the control disc. The plates were incubated for 72 h and imaged.

### Image analysis

Fiji [41] or Zeiss Zen software was used for image analysis. GraphPad Prism Software (Inc., San Diego CA, www.graphpad.com) was used for analysing data and making graphs. Inkscape software was used for preparing figures.

For the measurement of background autofluorescence in the hyphae expressing FtsZ-mTq2, 500×500 pixel (px) frames were extracted from three different time lapses of WT and LUV320 strains expressing FtsZ-mTq2 in regions of good focus, selected for in the DIC channel. 5×5 px circles were drawn over the well-focused hyphae in a beads-on-a-string fashion. The selections were overlayed onto the cyan fluorescent protein (CFP) channel image of the frames, and any circles falling on cross-walls were eliminated. The mean fluorescence in these circles was then extracted and pooled from all three (376 points from the *fbiC* mutant and 466 points from the WT) images and plotted.

## Results

### *S. venezuelae* exhibits autofluorescence characteristic of F_420_ in the CFP fluorescence channel

**Fig. 1.**
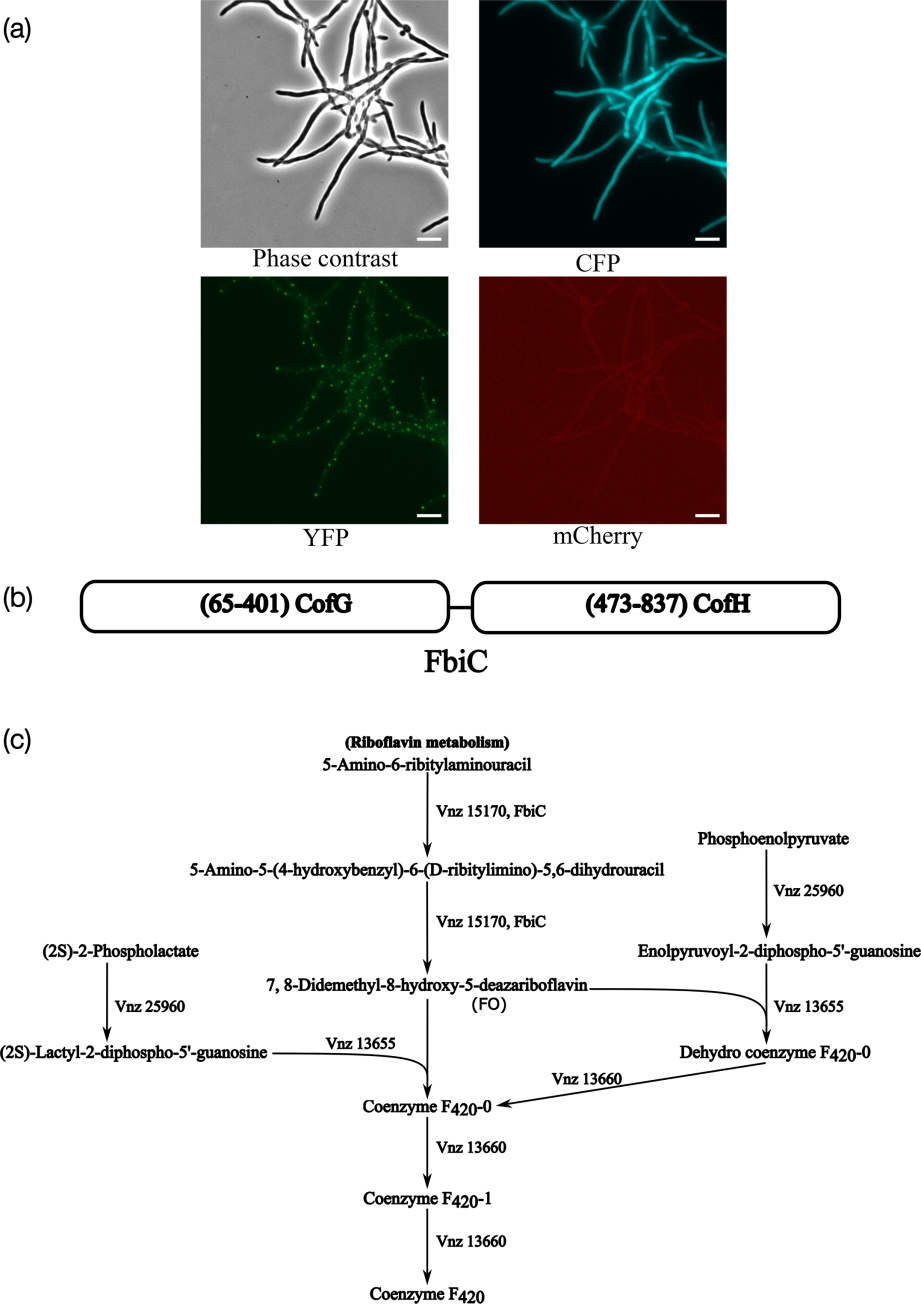
*S. venezuelae* exhibits autofluorescence in CFP and YFP channels. (a) Autofluorescence characteristics of WT *S. venezuelae* observed across fluorescence channels for imaging cyan (CFP), yellow (YFP) and mCherry fluorescent proteins. Fluorescence micrographs were captured with filter sets for CFP (excitation 424–448 nm; emission 460–500 nm), YFP (excitation 488–512 nm; emission 520–550 nm) and mCherry (excitation 559–585 nm; emission 600–690 nm) fluorescence channels. Representative images show the intensity distribution between the brightest and darkest pixels. Scale bar, 5 µm. (b) FbiC is a protein that consists of CofG and CofH domains and is predicted to catalyse the first steps in the F_420_ biosynthesis. (c) Predicted genes involved in the F_420_ synthesis pathway in *S. venezuelae*, including *fbiC* (*cofGH; vnz15170*), *cofD* (*vnz13655*), *cofE* (*vnz13660*) and *cofC* (*vnz25960*), based on KEGG pathway database analysis. A search for F_420_ biosynthesis-related genes in *S. venezuelae* shows the predicted metabolic network. Genes were identified from the pathway and used to draw the depicted table.

We examined the autofluorescence characteristics of the WT *S. venezuelae* across routinely used fluorescence channels for imaging using cyan (CFP: excitation 424–448 nm; emission 460–500 nm filter set), yellow (YFP: excitation 488–512 nm; emission 520–550 nm filter set) and red (mCherry: excitation 559–585 nm; emission 600–690 nm filter set) fluorescent proteins. Notably, we observed distinctive autofluorescence patterns in both the cyan (CFP) and yellow fluorescent protein (YFP) channels. The strain exhibited characteristic localized punctate fluorescence in the yellow range (Fig. 1a). The autofluorescence in the green and yellow channels has been noted earlier in *S. coelicolor*, possibly arising from the same source as the spectra of green fluorescent protein and YFP overlap significantly [24,25]. This autofluorescence has relatively weak intensity, but due to its irregular and spotty distribution, it is likely to interfere with weak signals that can be overwhelmed by this background. The basis of this autofluorescence in the green/yellow range is not well characterized.

Conversely, a relatively intense and diffuse autofluorescence could be observed in the CFP channel. Commonly used CFP-derived fluorescent proteins that are useful for fluorescence-tagging [42], as well as the most reliable FRET pair derivatives of the CFP–YFP protein pair [42], are likely to be hindered by this strong intrinsic fluorescence.

A possible origin of the cyan autofluorescence could be the prominent fluorescent molecule coenzyme F_420_. The biosynthesis pathway for F_420_ is widely distributed among *Actinomycetota* [43]. F_420_ emits blue–green fluorescence when excited with 420 nm light. The biosynthetic pathway for coenzyme F_420_ is well understood in mycobacteria, and the F_420_-associated autofluorescence has been successfully eliminated through rational genetic manipulation of the associated genes. F_420_ is derived from riboflavin [44]. In *Mycolicibacterium smegmatis*, F_420_ synthesis involves genes *cofG-cofH* (*fbiC*), *cofC (fbiD*), *cofD* (*fbiA*) and *cofE* (*fbiB*) [45]. Its synthesis is initiated by the conversion of 5-amino-6-ribitylaminouracil by the FbiC protein in a two-step process, leading to the production of F_O_ (Fig. 1b). F_O_ is then converted to co-factor F_420_ enzymatically by FbiA/CofD, FbiD/CofC and FbiB/CofE proteins. Co-enzyme F_420_ has a fluorescence excitation maximum of 420 nm and an emission maximum between 470 and 480 nm. A KEGG [31] search for the pathway of F_420_ synthesis in *S. venezuelae* shows the presence of the genes predicted to be involved in the F_420_ synthesis (Fig. 1c), namely *cofG-cofH* (*fbiC*): *vnz15170*, *cofD: vnz13655*, *cofE: vnz13660* and *cofC: vnz25960*. Based on a publicly available global transcription start site mapping data set in *S. venezuelae* [46], *fbiC* is expressed throughout the developmental life cycle of *S. venezuelae*, possibly in an operon with *vnz15175*, which encodes a putative F_420_-dependent oxidoreductase (Fig. S1). *cofC* appears to be in an operon with a 77 amino acid hypothetical protein (Fig. S2), while *cofD* and *cofE* appear to be co-transcribed from a single promoter (Fig. S3).

**Fig. 2.**
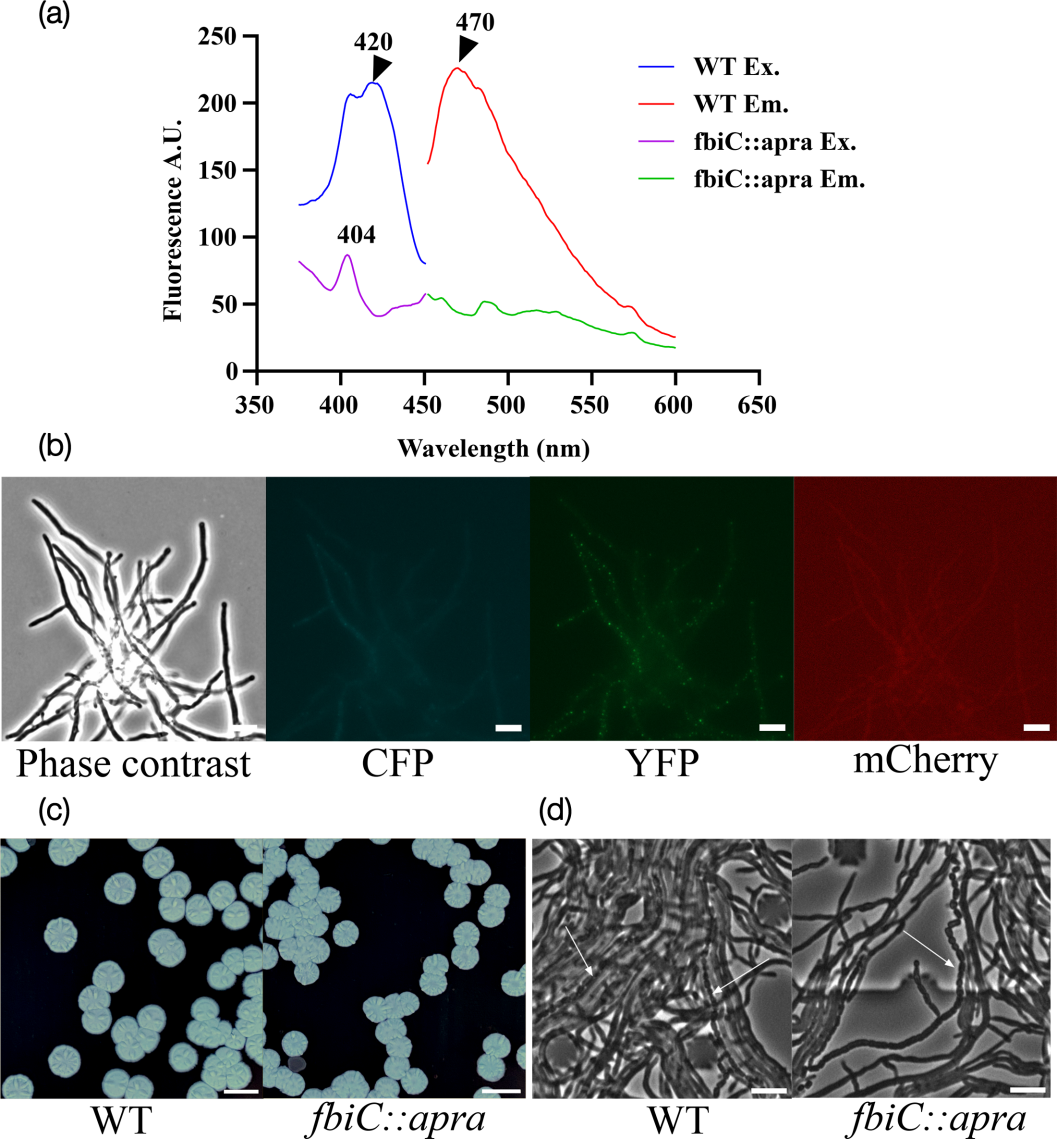
*fbiC* deletion leads to the elimination of autofluorescence in CFP channels without affecting cell growth and development. (a) Spectral analysis and characterization of autofluorescence in *S. venezuelae* WT and *fbiC* mutant strains. Excitation (Ex.; emission set to 475 nm) and emission (Em.; excitation set to 420 nm) spectra are shown of cell extracts from WT *S. venezuelae* and *fbiC* mutant strain LUV320. (b) Wide-field fluorescence microscopy images of *fbiC* mutant captured with filters for CFP, YFP and mCherry fluorescence, respectively (representative image shows fluorescence distribution between the brightest and the darkest pixel). (c) Colony morphology and pigmentation of LUV320 and WT on MYM agar medium after 3 days of incubation at 30°C. Scale bar, 5 mm. (d) Images from live-cell imaging (Movie S1). WT and LUV320 strains were grown in a microfluidic cell perfusion system as described in the ‘Methods’ section. Phase contrast imaging of the growing cells was performed, and representative frames were extracted after sporulation. Arrows point to spore chains. Scale bar, 5 µm.

Based on the existence of the F_420_ synthesis pathway, we hypothesized that it could be the source of autofluorescence in the CFP channel in *S. venezuelae*. To test this hypothesis, we examined the spectral characteristics of cell extracts generated from vegetatively growing *S. venezuelae* by measuring the excitation spectrum for the CFP range of fluorescence emission (470±5 nm) using fluorescence spectroscopy. The spectrum revealed an excitation peak around 420 nm (Fig. 2a), and a corresponding emission spectrum revealed a peak close to 470 nm (Fig. 2a). These spectra are highly similar to F_420_ [47,48] and support the hypothesis, thereby allowing for a rational strategy for eliminating autofluorescence by targeting F_420_ biosynthesis. *fbiC* (*vnz15170*) is the first gene in the synthesis pathway of F_420_ and is the ideal target (Fig. 1c). To test whether F_420_ is the source for autofluorescence in *S. venezuelae*, we deleted *fbiC* via homologous recombination to create the Δ*fbiC::apra* strain (LUV320) and a marker-less deletion strain (Δ*fbiC::FRT*, LUV336). Measurements of emission and excitation spectra in cell extracts of the LUV320 mutant and comparison to those of the WT strain revealed a substantial reduction of fluorescence in the spectral region expected for F_420_-dependent fluorescence (Fig. 2a). With excitation at 420 nm, the WT strain has a strong fluorescence in the range of 450 to 500 nm, with the emission maximum around 470 nm. In the LUV320 strain, this signal was found to be absent with excitation at 420 nm. The excitation spectrum with emission at 470 nm revealed a minor peak at 404 nm from an unknown source. In summary, the data show that autofluorescence in the cyan range of the spectrum in *S. venezuelae* can largely be ascribed to F_420_ and/or F_O_ and can be diminished by the deletion of *fbiC*. Interestingly, WT *S. venezuelae* mycelium exhibits a background of autofluorescence not only intracellularly, but also in the spent medium (Fig. S4). The majority of this autofluorescence is also lost upon deletion of *fbiC*. The origin of this autofluorescence is possibly from Fo, which is known to diffuse out of the cells into the medium due to its neutral charge [49,50].

Next, we compared the cellular autofluorescence signal of the LUV320 strains by wide-field fluorescence microscopy in the channels of interest (CFP, YFP and mCherry). We found that excitation of the LUV320 cells with the CFP filter set showed drastically reduced diffuse autofluorescence (Fig. 2b). The autofluorescence in the YFP and mCherry channels remains unaffected by the mutation. These results suggest that *fbiC* mutant strains (like LUV320 and LUV336) should be better suited for fluorescence imaging with CFP-tagged proteins than the WT.

### Abolishing F_420_ biosynthesis does not detectably affect the growth, development or oxidative stress tolerance

**Fig. 3.**
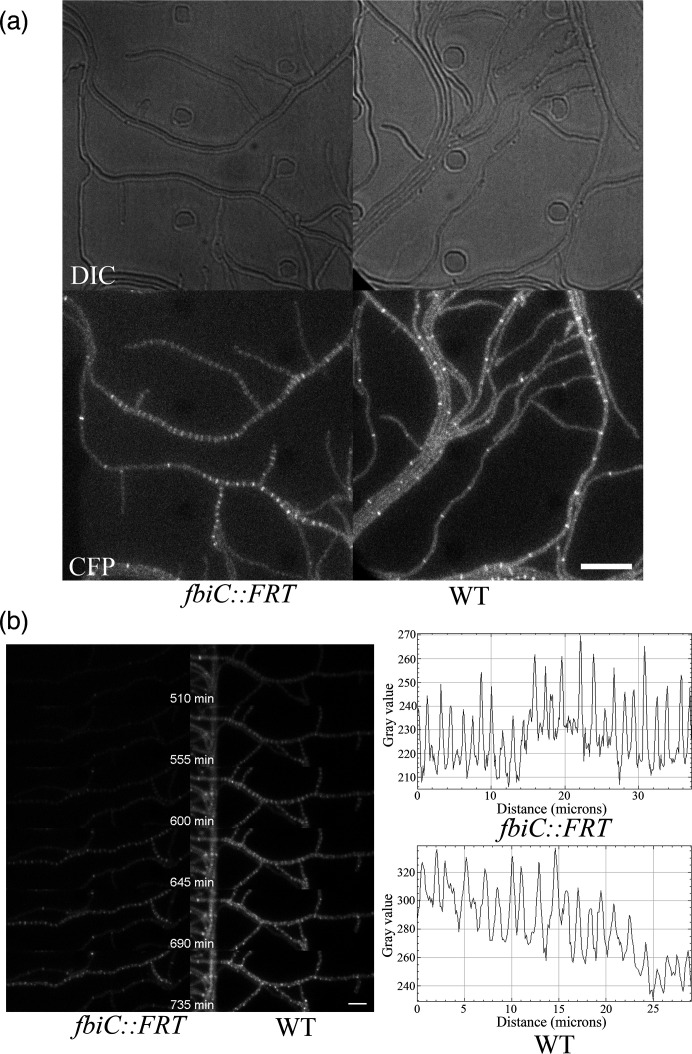
Live imaging of FtsZ-mTq2 localization during sporulation in *S. venezuelae*. (a) Representative fluorescence micrographs depicting the formation of FtsZ rings after the induction of sporulation. Fluorescence signal from FtsZ-mTq2 rings in the *fbiC* mutant strain LUV336 and WT *S. venezuelae* are shown below the corresponding DIC image. Cells were grown from spores to hyphae for 6 h in MYM medium in a microfluidic cell perfusion system before switching to spent MYM medium to induce sporulation. pent MYM from the WT and LUV320 strains was used, respectively. The representative micrographs were obtained at the 10 h time point of time-lapse live imaging (Movie S4). Scale bar, 10 µm. **b**. Close-up micrographs showing regions of FtsZ ring formation in the LUV336 and WT strain. Scale bar, 5 µm. The time points (min) of the experiment are indicated. Examples of the representative intensity profile of the Z-ring ladders at 630 min are shown to the right. The clearer peaks of FtsZ rings in the LUV320 background highlight the improved signal-to-noise ratio achieved by eliminating autofluorescence from coenzyme F_420_.

For the utility of the strains in physiological studies, *fbiC* deletion should not drastically affect the growth, morphology or developmental life cycle of the strain, which we examined next. LUV320 displayed normal colony morphology in MYM agar medium (Fig. 2c). We observed normal pigmentation of the colonies, indicating normal sporulation. Further, we performed live cell imaging of the WT and the mutant strains over the developmental life cycle from vegetative growth to sporulation. The mutant strain produces spores and shows similar morphology compared to the WT (Fig. 2d, Movie S1 and FtsZ localization in Fig. 3), showing that the developmental cycle of the *fbiC* mutant is not detectably affected when observed on rich MYM medium. However, it cannot be excluded that the effects of *fbiC* vary with different growth media or more stressful conditions.

F_420_ serves as a redox co-factor in a diverse but not yet fully characterized set of reactions in bacteria. Therefore, the *fbiC* mutation could potentially sensitize the cells to redox stressors. To test this possibility, we examined the LUV320 response to reductive-oxidative stress. We subjected LUV320 and WT to oxidant diamide and reductant DTT via disc diffusion assay and observed similar zones of clearance after 3 days of incubation, indicating no drastic detrimental effects of the mutation in response to these stressors (Fig. S5). DTT and diamide, however, affect cellular redox balance in a limited way. Further investigations of possible effects on redox status would be needed to fully establish the effect of deletion of *fbiC* on the cells.

### Reduced autofluorescence in *fbiC* mutants facilitates the determination of subcellular localization of CFP-tagged proteins in *S. venezuelae*

**Fig. 4.**
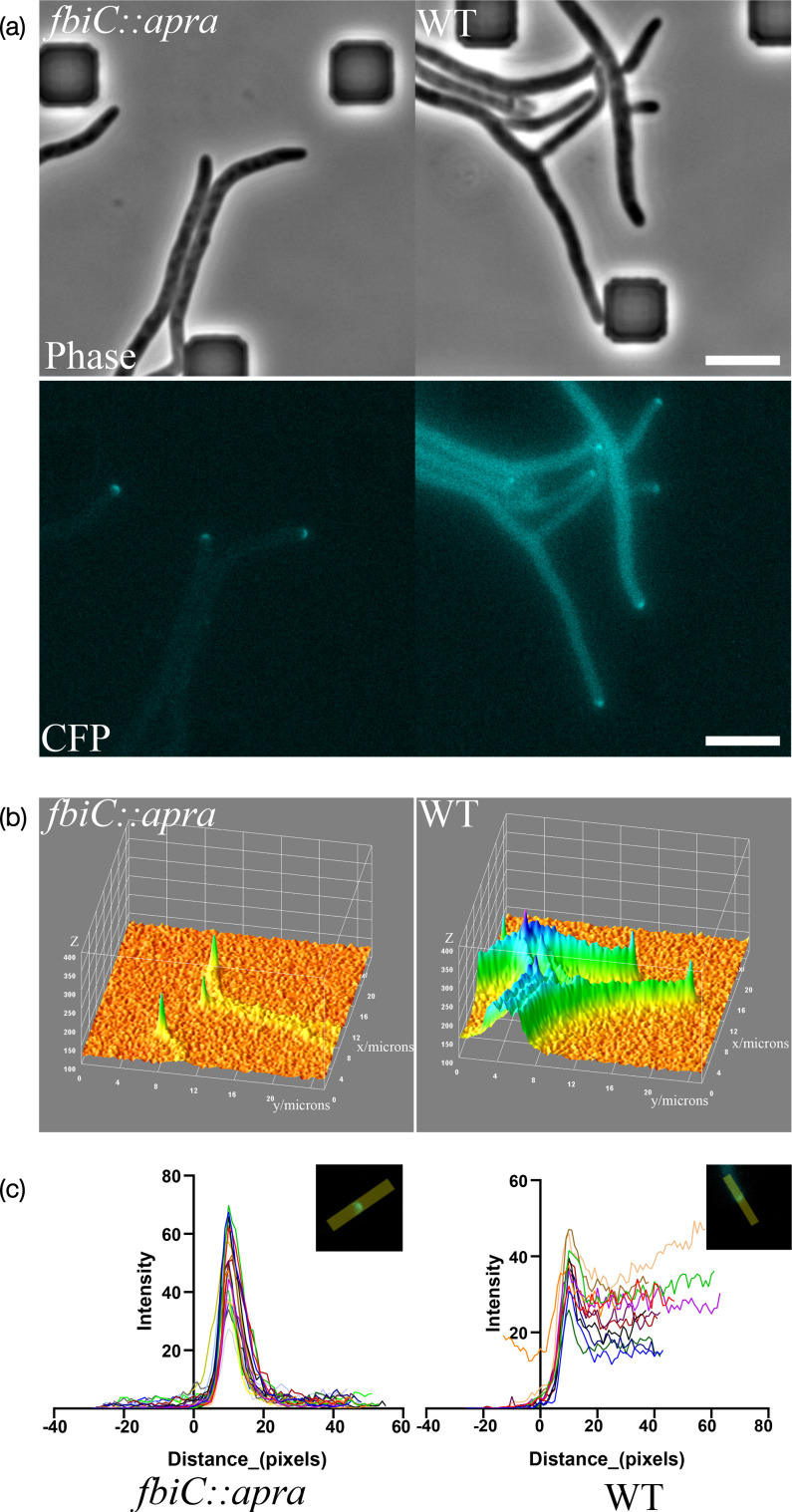
Effect of *fbiC* deletion on visualization of mTq2-tagged DivIVA in *S. venezuelae*. (a) Representative fluorescence micrographs from time-lapse imaging of WT and *fbiC* mutant strain of *S. venezuelae* expressing DivIVA tagged with mTurquoise2 (DivIVA-mTq2) during vegetative growth stage (Movie S2). Scale bar, 5 µm. (b) 3D surface plot of the pixel intensity of the grayscale CFP channel from panel (a). The fluorescence data from panel (a) was plotted using a 3D surface plot in Fiji software. The *x-* and *y-*axes are each 25 µm in length, and the *z*-axis represents the fluorescence intensity in the range same as panel (a) (100–400). (c) Plot showing fluorescence intensity profiles DivIVA-mTq2 along the growing hyphae. The graphs show data from 20 hyphal tips in *fbiC* mutant strain LUV320 and 12 tips in WT background. Using Fiji software, a line of 10-pixel width was drawn across the cell tip, and the intensity profile was plotted. The peaks of the fluorescence were aligned at the maximum, and the *x*-axis represents the fluorescence profile from outside the cell to the inside. The *y*-axis is set to 0 at 10 pixels from the signal maximum. Insets show a representative line used to plot the intensity. The data was analysed in GraphPad (Prism) software.

To demonstrate the usefulness of the *fbiC* mutant strains for imaging in the CFP channel, we next tested the quality of fluorescence signal in two different stages of the growth cycle, the vegetative stage, using DivIVA tagged with mTurquoise2 (mTq2) and the sporulation stage using FtsZ tagged with mTq2. Signals from weakly expressed genes can be easily overwhelmed by the background from the autofluorescence from F_420_. Although DivIVA is expressed abundantly in the cells, the expression constructs for fluorescent protein fusion to DivIVA that we use are relatively lower yielding, in order to avoid negative effects on cell shape and morphology [51]. As observed in Fig. 4 (Movie S2), the autofluorescence in the WT hyphae severely hinders the detection of the DivIVA-mTq2 signal. However, the deletion of *fbiC* leads to loss of the autofluorescence from F_420_, allowing for distinct visualization of protein localization. The quality of the fluorescence signal increases in the *fbiC* mutant (Fig. 4), vastly improving the detection of mTq2-tagged proteins in the imaging of low-abundance proteins. Further, DivIVA-mTq2 can be combined with the membrane staining dye FM4-64 to allow monitoring of the localization of the protein with respect to the cell membrane with minimal spectral overlap (Fig. S6).

Next, we examined the localization of proteins related to cell division. We used mTq2-tagged FtsZ to visualize its dynamics in sporogenic hyphae. To induce sporulation in *S. venezuelae* mycelium growing in the microfluidic cell perfusion system, spent medium was pumped into the microfluidic growth chambers after vegetative growth. Since the spent MYM medium for WT shows autofluorescence, we wondered how this would affect the autofluorescence in the LUV320 strain. We tested this by following the growth of the LUV320/DivIVA-mTq2 strain. After the initial growth on fresh MYM medium, we induced sporulation in the growing hyphae using spent MYM from the WT strain. We found that the mutant strain acquires autofluorescence upon switch from fresh to spent medium derived from the WT strain and reduces the quality of the signal (Fig. S7, Movie S3). This is relevant when studying sporulation stages of the cell cycle of *S. venezuelae*, where spent MYM is essential for sporulation induction. We looked at the localization of FtsZ-mTq to compare the signal between the WT and *fbiC* mutant strains. We compared the signal quality of FtsZ-mTq2 in WT and *fbiC* mutants when using spent MYM coming from respective parental strains. Upon induction of sporulation in the WT strain, the F_420_-derived autofluorescence interferes with imaging of FtsZ localization, while in the *fbiC* deletion strain, after induction with spent medium from an *fbiC* mutant, the background fluorescence signal from hyphae is visibly lower, leading to better signal-to-background ratio (Fig. 3a and b, Fig. S8, Movie S4). In summary, the *fbiC* mutant strain is useful for studies of subcellular localization of proteins tagged with CFP-like fluorescent proteins in both vegetative and sporulation stages of the developmental life cycle.

## Discussion

The complex secondary metabolism of streptomycetes leads to the production of fluorescent molecules that can hinder fluorescence-based imaging. Our investigation demonstrated F_420_-related autofluorescence in *Streptomyces*. Based on this finding, we deleted *fbiC* in *S. venezuelae* (*vnz15170*)*,* resulting in a strain depleted of autofluorescence with excitation around 420 nm and emission around 470 nm, i.e. in the range suitable for cyan fluorescent proteins. The strains developed in this work widen the toolbox of useful fluorescent proteins for studying *S. venezuelae*.

F_420_ was first isolated from the methanogenic archaea *Methanobacterium* strain M.o.H [47,52]. F_420_ is synthesized from F_O_ as the precursor [14]. F_O_ is the antennal chromophore that is the universal DNA photolyase cofactor. F_420_ fluorescence has an excitation maximum of 420 nm, which can be reduced by dithionite and NaBH_4_. The reduced F_420_ was found to be non-fluorescent, while the oxidized form exhibited strong fluorescence. Biochemically, F_420_ has a relatively low redox potential of −340 mV under standard conditions. The reduced form of F_420_ is involved in methanogenic reactions in *Methanobacterium*. TSS data from *S. venezuelae* show that F_420_ synthesis-related genes are expressed actively through the cell cycle, and the strong autofluorescence indicates its abundant synthesis in the cells of *S. venezuelae*.

F_420_ is considered a rare cofactor and has been thought to be synthesized mainly by *Euryarchaeota* and *Actinomycetota*. However, genome data mining has indicated that F_420_ is synthesized broadly among soil-dwelling aerobic bacteria such as alphaproteobacteria [53,54]. It is synthesized universally by mycobacteria. The function of the molecule is diverse. In mycobacteria, F_420_ plays an important role in facilitating metabolic flexibility due to its low redox potential [55]. The co-factor has been noted to be involved in antibiotic synthesis in some species [56]. In related *Mycobacterium* species, F_420_ is important for protection against oxidative stress [57]. While DTT or diamide-induced stress did not affect the survival of utant of *fbiC S. venezuelae*, a more comprehensive test under select redox stressors would be needed to reveal its specific function as a low redox potential cofactor. Given our results, the deletion of *fbiC* can improve the subcellular localization of CFP-tagged proteins with minimal effect on cell morphology.

The *fbiC* mutant strain also has a potential application for imaging with CFP-YFP fluorescence resonance energy transfer (FRET)-based biosensors. In the case of mycobacteria, F_420_ autofluorescence has been successfully removed by targeting F_420_ synthesis in *M. smegmatis*, allowing for successful application of the genetically encoded FRET-based biosensor ATeam1.03^YEMK^ [58,59]. Numerous FRET-based biosensors rely upon FRET between CFP and YFP pair, making this strain suitable for adaptation of these sensors for use in *Streptomyces*. For instance, FRET-based cyclic nucleotide messenger biosensors [60] can be adapted to *S. venezuelae* to study the dynamics of cyclic nucleotides with live imaging, some of which play important roles in cellular differentiation [61]. The low autofluorescence strain is also useful for multi-channel fluorescence imaging by broadening the useful range of fluorescent tags. For instance, the strain can be highly useful for studying the spatiotemporal dynamics of multiple proteins at the same time using multichannel imaging.

## Abbreviations

CFP: cyan fluorescent protein
DIC: differential interference contrast
DTT: dithiothreitol
F420: factor 420
FRET: fluorescence resonance energy transfer
KEGG: Kyoto Encyclopedia of Genes and Genomes
MYM: maltose yeast extract medium
YFP: yellow fluorescent protein
